# Complete Genome Sequence of *Burkholderia cepacia* Strain LO6

**DOI:** 10.1128/genomeA.00587-15

**Published:** 2015-06-11

**Authors:** Mahdi Belcaid, Yun Kang, Apichai Tuanyok, Tung T. Hoang

**Affiliations:** a Hawaii Institute of Marine Biology, Kaneohe, Hawaii, USA; b Department of Microbiology, University of Hawaii, Honolulu, Hawaii, USA; c Department of Infectious Diseases and Pathology, University of Florida, Gainesville, Florida, USA

## Abstract

*Burkholderia cepacia* strain LO6 is a betaproteobacterium that was isolated from a cystic fibrosis patient. Here we report the 6.4 Mb draft genome sequence assembled into 2 contigs. This genome sequence will aid the transcriptomic profiling of this bacterium and help us to better understand the mechanisms specific to pulmonary infections.

## GENOME ANNOUNCEMENT

The *Burkholderia cepacia* complex (BCC) is a group of closely related species which can occupy a diverse range of environmental niches and to transition into a potentially pathogenic lifestyle in plants, animals, and humans (1, 2). Cystic fibrosis (CF) patients are particularly prone to BCC infections, with patient-to-patient spread and high rates of morbidity and mortality (3). The *B. cepacia* LO6 strain was recovered from an infected CF patient, and belongs to the *B. cepacia* genomovar VI, a new member of the BCC (4, 5).

Here we present the full genome sequence of *B. cepacia* strain LO6. A single colony of strain LO6 that was grown on Luria Bertani (LB) agar plate was inoculated into LB and grown overnight at 37°C with a shaking speed of 225 rpm. Genomic DNA was isolated from the LO6 liquid culture using the Wizard genomic DNA purification kit from Promega (Madison, WI) and sequenced using the PacBio RS II system at the National Center for Genome Resources. The sequencing was performed using DNA/polymerase binding kit P5 and sequenced with PacBio DNA sequencing reagent 3.0. The high-throughput sequencing yielded 74,198 reads, totaling 346,270,766 bp.

The sequencing reads were assembled using the PacBio single-molecule real-time (SMRT) analysis software v2.3.0 into 2 high-quality contigs (chromosome I: 6,419,376 bp, plasmid: 4,304 bp), with 66.99% and 53.62% G+C content, respectively. The mean coverage for chromosome I and the plasmid was 42 and 985.8, and the consensus accuracy was above 99.993% for both. The resulting contigs were annotated using the Rapid Annotation using Subsystem Technology (RAST) server (6), which resulted in the identification of 6,064 coding sequences, including 67 tRNA and 17 rRNA coding sequences.

### Nucleotide sequence accession numbers.

This sequencing project has been deposited at GenBank under the accession numbers CP011301 and CP011302.
